# Lethal Nipah Virus Infection Induces Rapid Overexpression of CXCL10

**DOI:** 10.1371/journal.pone.0032157

**Published:** 2012-02-29

**Authors:** Cyrille Mathieu, Vanessa Guillaume, Amélie Sabine, Kien Chai Ong, Kum Thong Wong, Catherine Legras-Lachuer, Branka Horvat

**Affiliations:** 1 Inserm U758, Human Virology, Ecole Normale Supérieure de Lyon, IFR128 BioSciences Lyon-Gerland Lyon-Sud, University of Lyon 1, Lyon, France; 2 University of Malaya, Kuala Lumpur, Malaysia; 3 University of Lyon 1, ProfileXpert, Lyon, France; University of Kansas Medical Center, United States of America

## Abstract

Nipah virus (NiV) is a recently emerged zoonotic Paramyxovirus that causes regular outbreaks in East Asia with mortality rate exceeding 75%. Major cellular targets of NiV infection are endothelial cells and neurons. To better understand virus-host interaction, we analyzed the transcriptome profile of NiV infection in primary human umbilical vein endothelial cells. We further assessed some of the obtained results by *in vitro* and *in vivo* methods in a hamster model and in brain samples from NiV-infected patients. We found that NiV infection strongly induces genes involved in interferon response in endothelial cells. Among the top ten upregulated genes, we identified the chemokine CXCL10 (interferon-induced protein 10, IP-10), an important chemoattractant involved in the generation of inflammatory immune response and neurotoxicity. In NiV-infected hamsters, which develop pathology similar to what is seen in humans, expression of CXCL10 mRNA was induced in different organs with kinetics that followed NiV replication. Finally, we showed intense staining for CXCL10 in the brain of patients who succumbed to lethal NiV infection during the outbreak in Malaysia, confirming induction of this chemokine in fatal human infections. This study sheds new light on NiV pathogenesis, indicating the role of CXCL10 during the course of infection and suggests that this chemokine may serve as a potential new marker for lethal NiV encephalitis.

## Introduction

Nipah virus (NiV) is a recently emerged zoonotic pathogen of the family Paramyxoviridae that is distinguished by its ability to cause fatal disease in both animals and humans. NiV along with Hendra virus (HeV) comprise the new genus Henipavirus (reviewed in [1]). NiV was first identified during an outbreak of severe encephalitis in Malaysia and Singapore in 1998–99, with pigs serving as the intermediate amplifying host [2]. Since 1998 NiV has caused regular outbreaks, primarily in Bangladesh and India, with the most recent occurrences at the beginning of 2011 [3]. In the majority of subsequent spillover events, the mortality rate among humans has been higher (∼75%) along with evidence of multiple rounds of person-to-person transmission [4], [5], [6].

The endothelial cells represent one of the major targets of NiV infection, which is characterized by a systemic vasculitis and discrete parenchymal necrosis and inflammation in most organs, particularly in the central nervous system (CNS). The high pathogenicity of NiV infection appears to be primarily due to endothelial damage, syncytia and vasculitis-induced thrombosis, ischemia and vascular microinfarction in the CNS, allowing the virus to overcome the blood-brain-barrier (BBB) and to subsequently infect neurons and glia cells in the brain parenchyma [2], [7].

Pathogenesis of NiV infection is still poorly understood. As the endothelium forms the primary barrier of the circulatory system, endothelial dysfunction during infection could broadly affect immune cell function by regulating cytokines, chemokines and cell receptors and influencing vascular permeability. To gain new insights into virus-host interaction, we analyzed the transcriptome signature of NiV infection in primary human umbilical vein endothelial cells (HUVECs). Among the ten most strongly upregulated genes 8 h after NiV infection, we identified CXCL10 (interferon gamma-induced protein 10, IP-10), a chemokine that promotes leukocyte trafficking by acting on T lymphocytes, NK and dendritic cells via its receptor CXCR3 [8]. In addition to its protective role during the viral infection, CXCL10 may enhance the severity of virus infection and cause neuronal apoptosis and calcium dysregulation [9], [10] and enhance autoreactive lymphoproliferation and brain injury [11]. We have confirmed secretion of CXCL10 by NiV-infected HUVECs by ELISA and showed that increased production of CXCL10 follows NIV replication in experimentally infected hamsters. Finally, we demonstrated production of CXCL10 in brain endothelial cells of patients with fatal acute NiV encephalitis. Altogether, these results suggest that CXCL10 may be an important regulator of NiV-induced pathogenesis as well as a potential marker for NiV infection.

## Results

### Primary human endothelial cells are highly permissive to Nipah virus infection

**Figure 1:**
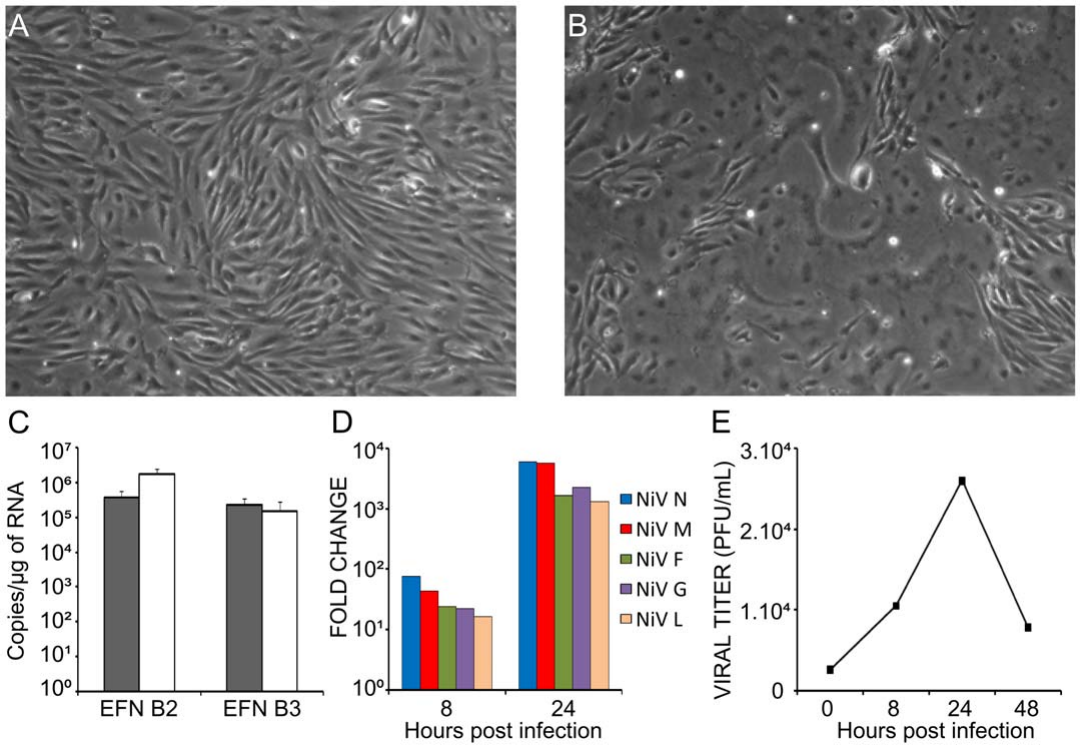
Primary human endothelial cells are highly permissive to NiV-infection. A, Mock-infected and B, NiV-infected HUVECs (MOI = 1) observed 24 h after infection show extensively developed syncytium formation. C, RT-qPCR analysis of the expression of NiV receptors ephrinB2 (EFNB2) and ephrinB3 (EFNB3) in HUVECs (black bars) and U373 astroglioma cells (white bars). D, Production of RNA specific for NiV genes: nucleoprotein (N), matrix (M), fusion protein (F), glycoprotein (G) and polymerase (L) in HUVECs during infection, determined by RT-qPCR. Fold change is relative to the number of copies of viral mRNAs 8 h or 24 h post infection compared to the number of copies obtained after 1 h of contact with the virus. E, Production of infectious NiV particles during the course of infection; supernatants taken at different time points were titrated on a Vero cell monolayer.

As endohelial cells are the first target of NiV infection, we have initially studied a permissivity of HUVEC cultures to NiV infection. We established the HUVEC cell culture and assessed their purity at passage 2, either by cell immunostaining or flow cytometry using two endothelial-specific markers, Von Willebrand Factor (VWF) stored in cytoplasmic Weibel-Palade bodies, and CD31 (PECAM-1) localized in homophilic intercellular contacts, which gave us reproducibly satisfactory results (Fig. S1). We then analyzed the expression of NiV receptors, production of viral specific RNAs, generation of the cytopatic effect and production of viral particles (Fig. 1). The expression of mRNA specific for NiV entry receptors ephrinB2 and B3 [12], [13], [14] was readily detected in these cells, at levels close to those in the astroglioma cell line U373, which was also highly permissive to NiV infection [15] (Fig. 1C). Production of NiV structural genes coding for nucleoprotein, matrix, fusion protein, glycoprotein and polymerase increased rapidly during the infection, following a gradient characteristic of paramyxoviruses, with highest expression of the first gene represented in the genome, N, and lowest for the gene coding for the polymerase (Fig. 1D). Formation of large syncytia was rapidly detected in HUVECs (Fig. 1B) and the highest level of the production of infectious particles was obtained 24H after infection, as most of the cells were lysed 48 h p.i. in this cell type (Fig. 1E). Therefore, NiV infects rapidly HUVECs and induces a significant cytopathic effect.

### Microarray analysis of NiV infection in HUVECs

**Figure 2:**
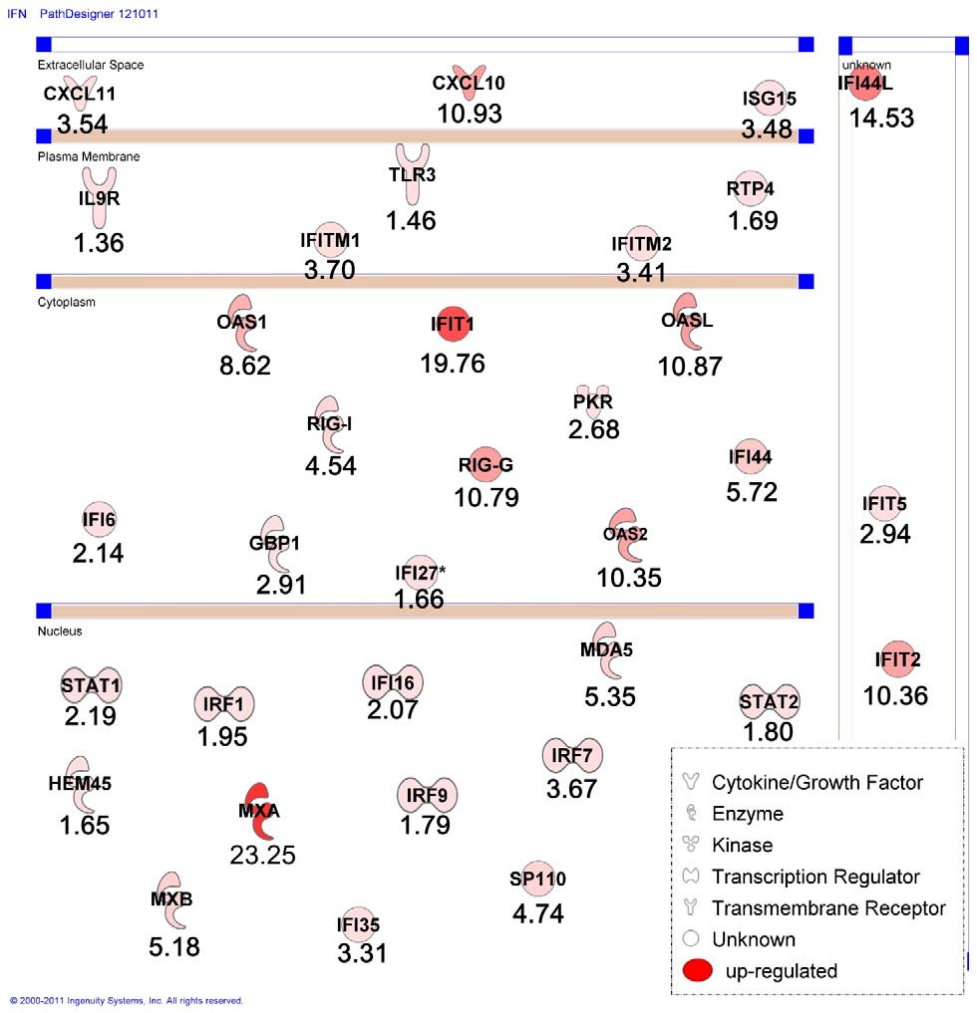
Impact of NiV infection on interferon pathway in HUVEC cells, determined using Ingenuity Pathway Analysis. NiV-induced upregulated genes are presented grouped, depending the cell compartment in which the corresponding proteins a localized (nucleus, cytoplasm, plasma membrane and extracellular space, for secreted proteins). Level of FC increase is indicated bellow each gene and intensity of red color corresponds to the FC.

**Figure 3:**
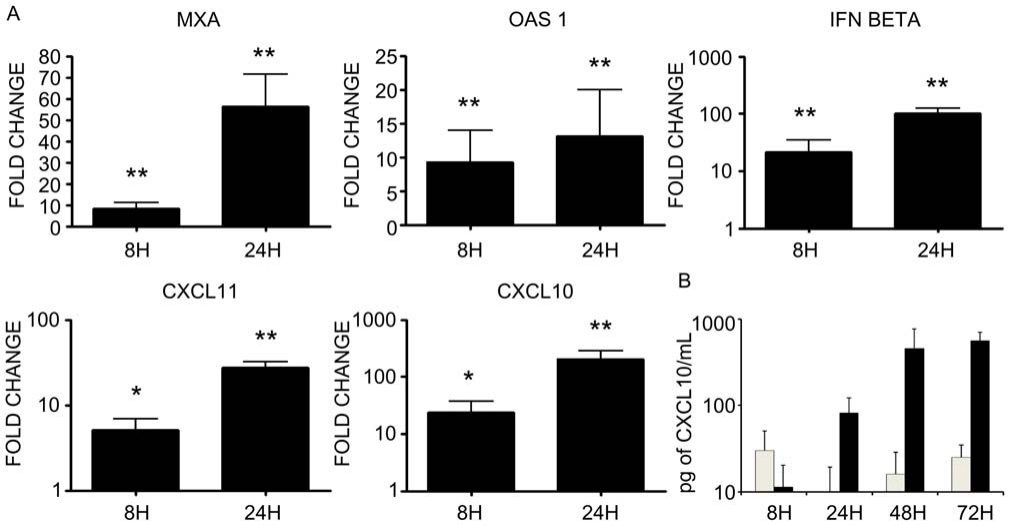
Analysis of expression of interferon-related genes in NiV-infected HUVECs. A, RT-qPCR analysis of the expression of MXA, OAS1, IFN beta, CXCL11 and CXCL10. B, ELISA analysis of the production of CXCL10 protein in mock-infected (white bars) and NiV-infected (black bars) HUVC cultures. Results are expressed as the average of 2–3 individual experiments and bars represent standard deviation. *p<0.05, **p<0.01, Mann-Whitney U-test.

To better understand virus-host interaction, we have analysed the effect of NiV infection at the level of gene expression in HUVECs, using Codelink microarray (see Materials and Methods). Cells were infected with either NiV or treated with mock preparation and RNA was taken 8 h p.i., to obtain the information about the early changes in NiV-induced gene expression in HUVEC in the conditions when cell viability was still preserved. Among 55.000 analyzed genes, pair-wise comparisons between infected and uninfected samples revealed 807 deregulated genes (fold change cut-off = 1.3). NiV infection down-regulated galectin 3 gene expression, a member of the lectin family, involved in cell adhesion, cell activation and chemoatraction [16], but most of the other down-regulated genes were not found associated with any known function (table 1). The 538 up-regulated genes were classified according to their Gene Ontology (GO) biological processes and molecular functions. This analysis revealed that NiV-infection up-regulated 34 genes implicated in the immune response, particularly those associated with the interferon pathway, including MxA, RIGI, MDA, 2′5′-OAS 1 and 2 (Fig. 2). Interestingly, among the top ten up-regulated genes was CXCL10 (interferon gamma-induced protein 10, IP10), an important chemokine secreted by endothelial cells (table 1). These results have been further confirmed by RT-qPCR at 8 and 24 h post-infection for several genes linked to interferon pathway, including MXA, OAS1, IFN beta and CXCL10 (Fig. 3A). In addition to stimulation of CXCL10, which was observed at different MOI of infection, MOI of 1 as well as 3, the induction of the closely related chemokine CXCL11 (Interferon-inducible T-cell alpha chemoattractant, I-TAC or IP-9), being at 19^th^ position among up-regulated genes, was also confirmed by RT-qPCR (Fig. 3A). However, the production of the third closely related chemokine CXCL9 (MIG), which shares the same receptor CXCR3 with CXCL10 and CXCL11, was not detected. Finally, secretion of CXCL10 by NiV-infected HUVECs was demonstrated by ELISA up to 72 h of infection (Fig. 3B), confirming the NiV infection induces the expression of CXCL10 at the protein level as well.

**Table 1:** Genes most highly induced or inhibited in HUVEC cultures 8 h after NiV infection.

FC	Common name	GenBank N°	Description	Function
23.3	PTPRM	AI669006	protein tyrosine phosphatase, receptor type, M	cellular growth and proliferation
23.2	MxA; IFI78; IFI-78K	AF135187	myxovirus (influenza virus) resistance 1, interferon-inducible protein p78	immune response
19.8	IFIT1; G10P1; IFI56; IFNAI1; GARG-16	NM_001548	interferon-induced protein with tetratricopeptide repeats 1	immune response
14.5	IFI44L; GS3686; C1orf29	NM_006820	interferon-induced protein 44-like	immune response
14.2	BRESI1	NM_033255	epithelial stromal interaction 1 (breast)	connective tissue disorder
11.6	MGC116903; MGC116904	NM_000811	gamma-aminobutyric acid (GABA) A receptor, alpha 6	neurological disease
11.4	cig5; vig1; 2510004L01Rik	NM_080657	radical S-adenosyl methionine domain containing 2	viral function
10.9	CXCL10; IFI10; IP-10	NM_001565	chemokine (C-X-C motif) ligand 10	immune response
10.9	OASL; TRIP14	NM_003733	2′-5′-oligoadenylate synthetase-like	immune response
10.8	RIG-G; IRG2; IFI60; ISG60; GARG-49	NM_001549	interferon-induced protein with tetratricopeptide repeats 3	immune response
10.4	IFIT2; G10P2; IFI54; GARG-39; ISG-54K	NM_001547	interferon-induced protein with tetratricopeptide repeats 2	immune response
10.3	OAS2; MGC78578	NM_002535	2′-5′-oligoadenylate synthetase 2, 69/71 kDa	immune response
8.6	OAS1	AU076579	2′,5′-oligoadenylate synthetase 1, 40/46 kDa	immune response
5.8	BAL; BAL1;	NM_031458	poly (ADP-ribose) polymerase family, member 9	cellular movement
5.7	IFI44; p44; MTAP44	NM_006417	interferon-induced protein 44	immune response
−5.4	LGALS3	AF031424	Homo sapiens galectin 3 (LGALS3) gene, exon 5.	immune response
−7.6	TAF4B	AA457205	TAF4b RNA polymerase II, TATA box binding protein ass factor, 105 kDa	cell signaling
−8	ATP13A4	AW073336	ATPase type 13A4	unknown
−10.3		BX108708	Transcribed locus	unknown
−12.3	IDS	AK126415	CDNA FLJ44451 fis, UTERU2023039	unknown
−13		BF476432	CDNA FLJ38408 fis, FEBRA2009029	unknown
−14.8		AI255115	Transcribed locus	unknown
−17.9	CTS9	NM_017418	deleted in esophageal cancer 1	cancer
−25.1	FLJ22318; DKFZp434K0926	NM_022762	hypothetical protein FLJ22318	unknown
−32.1	MGC3035	NM_024293	chromosome 2 open reading frame 17	unknown
−44	BBS4	BF370543	Bardet-Biedl syndrome 4	cancer
−46.7	FLJ12986	AK126695	hypothetical protein FLJ12986	unknown

**FC**: Fold Change; induction of the level of gene expression in NiV-infected cells in comparison to mock-infected cells as determined by microarray analysis. Only changes higher then 5.7 and lower then −5.4 are presented.

### Expression of CXCL10 mRNA follows NiV replication *in vivo*

**Figure 4:**
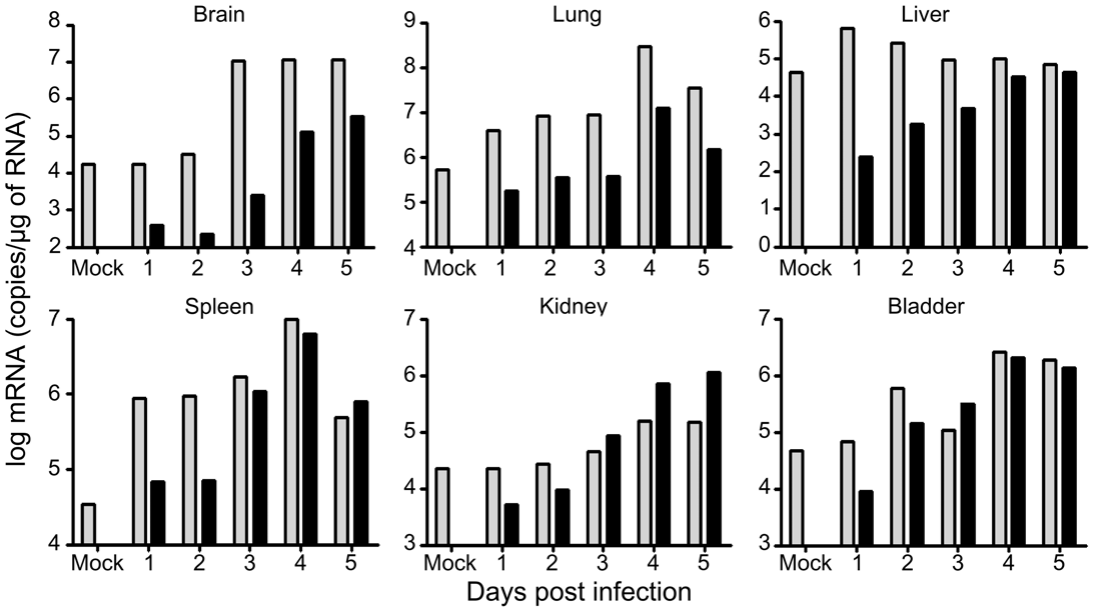
Expression of CXCL10 and NiV nucleoprotein (N) genes in organs obtained from NiV-infected hamsters during the course of infection. Hamsters were sacrificed at different time points after infection and RNA was isolated from different organs and analyzed by RT-qPCR. Grey bars correspond to CXCL10 and black bars to NiV-N, calculated as described in Methods. Significant correlation was found between expression of CXCL10 and expression of NiV N (R^2^ = 0,989, p<0,001, Pearson test).

As CXCL10 is known to have an important immunobiological function in the organism, which received a great deal of attention in recent years, we focused further studies on this chemokine. Thus, we analyzed whether NiV-infection induces production of CXCL10 *in vivo*, using the hamster animal model, which closely reproduces human infection and induces lethal outcome starting from day 5 p.i. [17]. Hamsters were infected by NiV and sacrificed on a daily basis for analysis of RNA in different organs (Fig. 4). The baseline expression of CXCL10 was observed in all organs and increased during the course of infection, particularly in brain and kidney. In lung and spleen the level of CXCL10 initially increased, but decreased on the last day that preceded lethal outcome of the infection. The most rapid and highest induction of CXCL10 mRNA expression was observed in the spleen (16 times more than the baseline expression, already 24 h p.i.), which may correlate with the abundance of cells capable of secreting CXCL10 in the spleen (leukocytes, endothelial cells and splenic stromal cells [18]. The highest NiV replication was observed in lungs of infected hamsters. The level of CXCL10 expression significantly correlated to the level of NiV N expression in analysed organs (p<0.001), suggesting that production of this chemokine closely follows the NIV replication.

### CXCL10 is highly expressed in the brain of NiV infected patients

**Figure 5:**
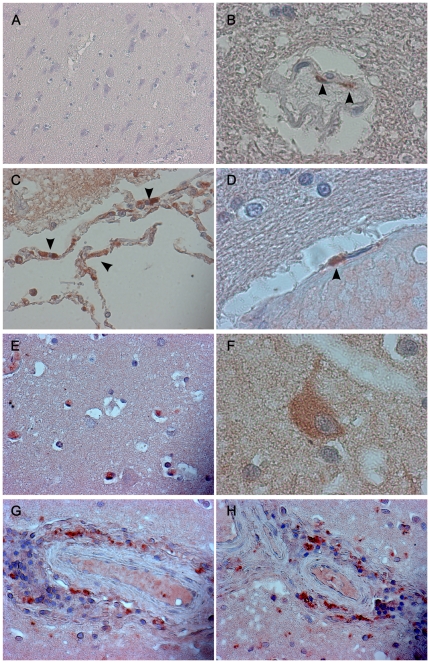
Immunohistochemical analysis of CXCL10 production in the brain durng NiV infection. Non-infected patients (A, ×100) and patients autopsied after lethal acute NiV-infection (B–H) were analysed for the expression of CXCL10 in endothelial cells in cerebral cortex (B, D, ×1000, arrows) and cells with neuronal morphology (E, F). CXCL10 immunoreactivity in perivascular inflammatory cells (C, G, H, ×400).

We have next performed immunohistochemical analysis of brain tissues from patients that succumbed to NiV-infection during the outbreak in Malaysia in 1999. Widespread vasculitis and perivascular infiltration were regularly detected after hematoxylin staining (Fig. 5). Staining for CXCL10 revealed production of this chemokine in brain endothelial cells (Fig. 5 B–F), as well as in perivascular infiltrating cells (Fig. 5 G–H) and in occasional cells with neuronal morphology (Fig. 5 E–F). CXCL10 expression was intense in the endothelium of cerebral vessels in encephalitic patients (Fig. 5 B–F) and was not observed in the cerebral cortex of non-infected patients (Fig. 5A). Altogether, these results indicated that NiV-infection activates cellular pathways leading to an increase of CXCL10 expression that may play a role in the pathogenesis of this highly lethal emergent infection.

## Discussion

The inflammatory cellular infiltrates found in the CNS of patients with acute NiV encephalitis include neutrophils, macrophages, lymphocytes and reactive microglia [7], which may all play an important role in NiV pathogenesis. Recruitment of these cells indicates a possible action of chemokines, known as main regulators of leukocyte trafficking. Our analysis of the transcriptome profile of NiV-infected primary endothelial cells revealed the induction of several genes involved in the interferon type I pathway as well as the overexpression of CXCL10, an important chemoattractant chemokine with proinflammatory activity [19]. Although expression of IFN alpha and beta was shown to be inhibited in human cell lines after NiV infection [20], human primary endothelial cells seem to be resistant to this inhibition [21]. Our study demonstrated that a few other members of interferon type I pathway, including MxA, RIGI, 2′5′-OAS 1 and 2, are highly expressed early after NiV infection, suggesting a functional interferon pathway in NiV-infected human endothelial cells.

CXCL10 is a secreted polypeptide of 10 kDa that was first identified as an early response gene induced after interferon gamma treatment in a variety of cells, and was thus named interferon gamma-inducible peptide, IP-10 [22], [23]. Furthermore, it was shown that interferon alpha could also induce CXCL10 in primary cultured neurons [24] and seminiferous tubules [25]. In addition to interferons, HIV envelope glycoprotein gp120 was shown to induce expression of CXCL10 in brains of mice [26]. HIV Tat protein can also induce CXCL10 in astrocytes [27]. Furthermore, CXCL10 is expressed early in the CNS in response to a wide variety of viruses [11], [13], [28], [29], [30]. Our results demonstrate that NiV-infection induces rapidly production of CXCL10 in primary endothelial cells. As production of interferon gamma by these cells is contested, it is possible that the generation of CXCL10 is either a direct effect of NiV proteins or is induced via NiV-activation of interferon beta, following infection. Moreover, our results demonstrate induction of CXCL10 *in vivo* in different organs of NiV-infected hamsters, as well as in patients that succumbed to lethal NiV-infection, thus revealing an important association between NiV-infection and CXCL10 production. Although endothelial cells were clearly secreting this chemokine, the perivascular inflammatory cells and some neurons are most probably participating in the production of CXCL10 as well.

Individual chemokines, including CXCL10, play opposing roles in neuroinflammation in different experimental models of infectious disease, making it difficult to predict whether they have a protective role by contributing to immune eradication of the microbial attack or they cause inflammatory damage and disease [19]. Therefore, potential effect of CXCL10 in NiV infection could be either beneficial or harmful and the balance between these two effects may be critical for the final outcome of the disease. Infection with RNA viruses that may cause encephalitis in humans, such as HIV or lymphocytic choriomeningitis viruses, or in rodent models, such as mouse hepatitis virus and Theiler's virus, can directly induce the expression of chemokines by astrocytes and microglia and establish chemokine gradients that promote leukocyte trafficking within the CNS [11], [31], [32], [33]. In addition, neutralization of CXCL10 decreased leukocyte migration to areas of infection in measles virus-infected brain tissue [34]. Although several of these viruses directly infect neurons, these cells have not been observed to participate in the inflammatory response, suggesting the indirect induction of inflammatory response via cytokines. Nevertheless, in a transgenic mouse model of measles virus encephalitis, neuronal expression of CXCL10 was associated with T-cell recruitment, suggesting that neurons may play a role in the induction of immune responses to viral invasion [35]. In recent study Lo *et al.* showed that NiV infection could induce production of CXCL10 and several other inflammatory chemokines in microvascular, but not in macrovascular endothelial cells [21]. However, in contrast to our study, they cultured endothelial cells in the presence of hydrocortisone, known to have important anti-inflammatory activity and to reduce the expression of pro-inflammatory cytokines [36], which could account to the differences with our results. In accord to our results, NiV was shown to induce CXCL10 expression as well as several other inflammatory chemokines in brain and lungs of NiV infected hamsters [37] suggesting altogether that the other cytokines may contribute to the CXCL10 proinflammatory effect.

CXCL10 has been detected in the cerebrospinal fluid of individuals with HIV-1 infection [38], [39] and in the brains of individuals with HIV-associated dementia [33], but was absent in uninfected control individuals. These authors also reported that CXCL10 levels were closely associated with the progression of HIV-1-related CNS infection and neuropsychiatric impairment. Moreover, CXCL10 and its receptor CXCR3 were shown to be present in the brains of macaques with SIV/SHIV-E [40], [41] and to elicit apoptosis in fetal neurons [9], [42]. The mechanisms of neuronal injury mediated by the CXCL10 was suggested to be associated to calcium dysregulation during CXCL10 mediated apoptosis [10], and we hypothesize that overexpression of CXCL10 in Nipah-infected patients may be directly involved in NiV-induced neuropathology. Lack of the appropriate regaents for hamster animal model prevented us from further *in vivo* analysis of the role of CXCL10 during Nipah infection.

CXCL10 is the substrate for serine protease CD26/dipeptidyl-peptidase 4, which cleaves its aminoterminal part and alters its receptor binding and signaling, producing therefore an antagonistic protein with dominant negative function [43]. This antagonist form of CXCL10 was very recently shown to play an important role in patients chronically infected with hepatitis C virus [44] and to present an important negative prognostic marker for the response to therapy [45], [46]. Whether this cleaved antagonist form of CXCL10 is generated during NiV-infection remains to be elucidated. If produced, its action on inhibition of the physiological role of CXCL10, may play an important role in the pathogenesis of NiV-encephalitis.

These results suggest that NiV-infection in endothelial cells induces CXCL10 production both *in vitro* and *in vivo* and highlight the use of molecular profiling of virus-infected cells as a powerful tool to define novel mechanisms of virus-host cell interaction. As equilibrium in cytokine production is essential in the generation of the adequate immune response, CXCL10 overexpression may be crucial for the development of NiV-associated encephalitis and could be a target for therapeutic approaches.

## Materials and Methods

### Ethics Statement

Umbilical cords were obtained between 2006 and 2008 from healthy full-term newborns with written parental informed consent according to the guidelines of the medical and ethical committees of Hospices Civils de Lyon and of Agence de Biomedecine, Paris, France. Ethics Committee approval for this study was not required according to institutional guidelines and French law N°2004-800, from 6 August 2004 – art 12 ORF 7.08.2008, Article L1245-2, allowing the utilization of the placenta and umbilical cords in scientific and therapeutic purpose when their donors do not express any opposition.

All animals were handled in strict accordance with good animal practice as defined by the French national charte on the ethics of animal experimentation. Animal work was approved by the Regional ethical committee (Comité Régional d'Ethique pour l'Experimentation Animal de la Région Rhone-Alpes, CREEA, protocol N° 220) and experiments were performed in the INSERM Jean Mérieux BSL-4 laboratory in Lyon, France (French Animal regulation comittée N° A69 387 05 02).

Autopsies of human brain tissue were performed after receiving written patient's relatives consent for autopsy studies at the Pathology Department of University of Malaya, Kuala Lumpur, Malaysia and their analysis was approved by review board of Faculty of Medicine of University.

### Cell culture

Primary HUVECs were isolated from human umbilical cords of 22 donors by treating the umbilical vein with 0.1% collagenase for 30 min at 37°C as described previously [47]. Cell cultures were pooled by sets of three donors for experiments. Cells were cultured in flasks coated with 0.2% gelatin in complete medium containing M199 medium (Gibco), 20% of fetal calf serum (Gibco), 100 µg/ml bovine brain extract [48], 14 mM Hepes (Gibco), 10 UI/ml heparin (Pfizer), and a cocktail of antibiotic/antimycotic (Gibco). At passage 4, cells were seeded at 30 000 cells/cm^2^ for 8 h, then serum deprived for 16 h prior to NiV-infection in complete medium. Immunofluorescent staining and analysis of HUVEC culture was performed as described in Methods S1. U373 astroglioma and Vero cells were maintained in DMEM medium (Invitrogen) supplemented with 10% fetal calf serum, 100 U/ml penicillin, 0.1 mg streptomycin, 10 mM HEPES and 2 mM L-Glutamine at 37°C in 5% CO_2_.

### Infection of HUVECs and titration

Nipah virus (isolate UMMC1, Genebank AY029767) [49] was prepared on Vero-E6 cells as described previously [50]. HUVECs were infected with a multiplicity of infection (MOI) of 1 and harvested for RNA isolation 8 and 24 hours post infection (p.i.). At indicated times p.i. 150 µl of cell culture supernatant were collected and frozen and viral titration was performed as detailed elsewhere [50].

### Infection of hamsters

Eight-week-old golden hamsters (Mesocricetus auratus, Janvier, France) were anesthetized and infected intraperitoneally (i.p.) with 0.4 ml of wild-type NiV (10 000 PFU) in the BSL-4 laboratory in Lyon. Each day post infection (p.i.), one hamster was euthanised and organs were frozen at −80°C.

### Microarray analysis

Total RNA was extracted from uninfected or NiV-infected HUVECs 8 h p.i., both prepared from two different pools, each containing 3 donors and cultured at same conditions, using RNeasy kit (Qiagen) according to the manufacturer's protocol. Quality of total RNA was checked on nanochips with the Agilent 2100 Bioanalyzer 2100 (Agilent Technologies,).

Amplified and biotin-labeled RNAs were obtained from 2 µg of total RNA, by a round of *in vitro* transcription (dIVT) using the Message Amp a RNA kit version II (Ambion), following the manufacturer's protocol. Different quantities of positive synthetic mRNA controls (spikes, corresponding to 6 bacterial RNAs, used to control sensitivity, quality of hybridization and data normalization) were added to all samples, during the first step of reverse transcription of total RNAs. Hybridization was performed on Codelink Uniset Human Whole Genome bioarrays (http://www.codelinkbioarrays.com). 10 µg of biotin-labeled RNA were fragmented using 5 µl fragmentation buffer in a final volume of 20 µl, then mixed with 240 µl Amersham hybridization solution and injected onto Codelink Uniset Human Whole Genome bioarrays, containing approximately 55.000 30-mer probes based on the NCBI/Unigene RefSeq database that permit the expression analysis of 57,347 transcripts and ESTs (GE Healthcare Europe GmbH). Arrays were hybridized overnight at 37°C, then washed in stringent TNT buffer at 46°C for 1 h before performing a streptavidin-cy5 detection step. Each array was incubated for 30 min in 3,4 ml streptavidin-cy5 solution, washed four times in 240 ml TNT buffer, rinsed twice in 240 ml water containing 0.2 M Triton X-100, and then, dried by centrifugation at 650 g. Arrays were then scanned at 635 nm using an Axon Genepix 4000B Scanner (Axon).

Data extraction and raw data normalization were performed using the CodeLink Gene Expression Analysis v4.0 software. Normalization was performed by the global method. The threshold was calculated using the normalized signal intensity of the negative controls supplemented by 3 times the standard deviation. Spots with signal intensity below this threshold were referred to as “absent”. Obtained datasets were deposited in GEO database in accord to MIAME guidelines (Accession number: GSE 32902: GSM813064, GSM813065, GSM813066 and GSM813067). Finally, data were converted to the excel format and data analysis was performed by using the Gene Spring v7.0 software from Agilent and pariwise comparisons were performed between infected and uninfected samples.

### Quantitative reverse transcription PCR (RT-qPCR)

Total RNA was extracted from mock and NiV-infected HUVECs 8 and 24 hours post-infection using RNeasy Mini Kit according to the manufacturer's instructions including additional step with RNase-free DNase (Qiagen). RNA was isolated from hamster organs 10–30 mg using a tissue lyser (Qiagen) in RLT buffer containing 1% ß-mercaptoethanol, according to manufacturer recommendations.

Reverse transcriptions were performed on 0.5 µg of total RNA using the Transcriptor first strand cDNA synthesis kit (Roche) and run in Biometra® T-GRADIENT PCR devise. Obtained cDNAs were diluted 1∶10. Quantitative PCR was performed using Platinum® SYBR® Green qPCR SuperMix-UDG with ROX kit (Invitrogen™). qPCR was run on the ABI 7000 PCR system (Applied biosystems) using the following protocol: 95°C 5′, and 40 cycles of 95°C 15″, 60°C 1′, followed by a melting curve up to 95°C at 0.8°C intervals. All samples were run in duplicate and results were analyzed using ABI Prism 7000 SDS software available in the genetic analysis platform (IFR128 BioSciences Lyon-Gerland). Glyceraldehyde 3-phosphate dehydrogenase (GAPDH) was used as housekeeping gene for mRNA quantification and normalization. GAPDH and standard references for the corresponding genes were included in each run to standardize results in respect to RNA integrity, loaded quantity and inter-PCR variations. Primers used were designed using Beacon 7.0 software, and validated for their efficacy close to 100% in control PCR amplification: EFN B2 for: TCGGGCTAGTTAAGGTGTGC, EFN B2 rev: ATGAGTGTTCCATGAGTGATGC, EFN B3 for: TCACCCTCTTGGCTTCTTATCC, EFN B3 rev: GGGGAGTGGTTGGTATGAGAG, NiV N for: GGCAGGATTCTTCGCAACCATC, NiV N rev: GGCTCTTGGGCCAATTTCTCTG, NiV L for: ATGGTGCTGTGCTGTCTCAGG, NiV L rev: AGCCGACATTTCTTGACAACCC, IFNß for: CTCCTAGCCTGTGCCTCTGG, IFNß rev: TGCAGTACATTAGCCATCAGTCAC, MxA for: AGCCACTGGACTGACGACTTG, MxA rev: AAATCACCACGGCTAACGGATAAG, OAS1 for: AGAACTTACCTCTTGCCAAAGG, OAS1 rev: GGACAAGGGATGTGAAAATTCC, CXCL10 for: GGAAGGTTAATGTTCATCATCCTAAGC, CXCL10 rev: TAGTACCCTTGGAAGATGGGAAAG, CXCL11 for: GGATGAAAGGTGGGTGAAAGGAC, CXCL11 rev: AACGTGAAAGCACTTTGTAAACTCC, GAPDH for: CACCCACTCCTCCACCTTTGAC, GAPDH rev: GTCCACCACCCTGTTGCTGTAG. Primers used for hamster study: hamster CXCL10 for: AGACAACAGTAACTCCAGTGACAAG, hamster CXCL10 rev: AGTGTAGCACCTCAGCGTAGC, murine GAPDH for: GCATGGCCTTCCGTGTCC, murine GAPDH rev: TGTCATCATACTTGGCAGGTTTCT. The relative expression represents the ratio of the number of copy of mRNA of interest versus mRNA of GAPDH and expressed in relationship to the quantity of RNA analyzed All calculations were done using the 2^ΔΔCT^ model of [51] and experiments were performed according to the MIQE guideline [52].

### ELISA analysis

Supernatants of mock and NiV-infected HUVECs were collected 8, 24, 48, 72 h p.i. and used to quantify the induction of CXCL10 using human IP-10 Immunoassay (Invitrogen™), according to manufacturer's instructions. All tests were performed on 2 sets of 3 donors, in duplicates.

### Immunohisotchemistry (IHC)

Autopsy brain tissue was obtained from 3 fatal acute Nipah cases from the outbreak in Malaysia in 1999, fixed in 10% buffered formalin and paraffin-embedded and from two control brain tissues from patients that succumbed to non-neurological diseases. Sections (4 µm) were deparaffinized with xylene and rehydrated through graded alcohol and distilled water. Antigen retrieval was performed in 10 mM trisodium citrate buffer, pH = 6, using waterbath (99°C, 20 min). Slides were then washed 3 times in PBS 1×. Endogenous peroxidase was blocked using H2O2, 3% in PBS, for 20 min at Room temperature (RT). Slides were then washed 2 times in PBS 1× and additional blocking was performed using goat serum (1∶20 in PBS) for 20 min at RT. Primary rabbit anti-human CXCL10 (Peprotech), was applied diluted at 4 µg/ml, overnight at 4°C. After 3 washes in PBS, slides were incubated with secondary goat anti-rabbit conjugated with HRP (Promega) 1∶500 for 1–2 hours at RT. Sections were then developed with AEC kit (VECTOR) for 25 min, washed in distilled water and counterstained with Harris hematoxylin solution (Sigma-Aldrich) 1∶3 in PBS, 30 s. After one wash in water (3 min) sections were mounted with DakoCytomation faramount aqueous mounting medium and coverslipped. Slides were analysed using Axioscope microscope equipped with Zeiss Axiovision software (Zeiss Geramny).

### Statistical analysis

Data were expressed as mean±standard deviation (SD). Statistic analyses were performed using Mann-Whitney U-test and Pearson correlation test.

## Supporting Information

Figure S1 **Expression of the endothelial-specific markers.** Von Willebrand Factor (VWF) and CD31 (PECAM-1) were analyzed in 2^nd^ day HUVEC cultures by immunostaining with anti-CD31 (green) and anti-VWF (red). Nuclei were stained with DAPI (blue). Analysis was performed as described in Methods S1.
(TIF)

Methods S1 **Supplementary methods.**
(DOCX)
