# Supplementary figures and images for: Lethal Nipah Virus Infection Induces Rapid Overexpression of CXCL10
Source: PLoS One. 2012 Feb 29;7(2):e32157. doi: 10.1371/journal.pone.0032157 (PMC3290546; doi:10.1371/journal.pone.0032157)

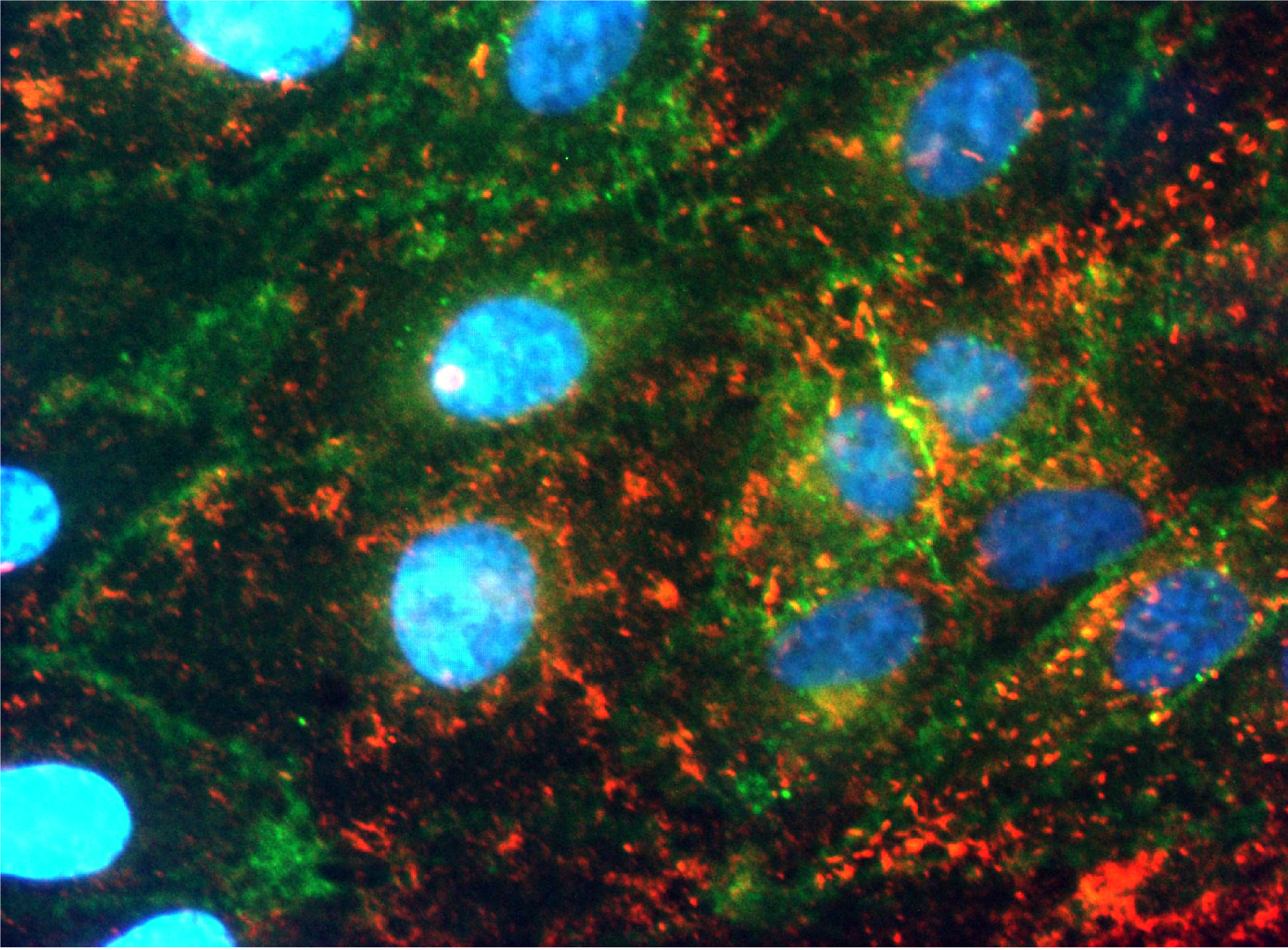

Supplement: Figure S1 — Expression of the endothelial-specific markers. Von Willebrand Factor (VWF) and CD31 (PECAM-1) were analyzed in 2nd day HUVEC cultures by immunostaining with anti-CD31 (green) and anti-VWF (red). Nuclei were stained with DAPI (blue). Analysis was performed as described in Methods S1. (TIF) [file pone.0032157.s001.tif]
