# Supplementary material for: Lethal Nipah Virus Infection Induces Rapid Overexpression of CXCL10
Source: PLoS One. 2012 Feb 29;7(2):e32157. doi: 10.1371/journal.pone.0032157 (PMC3290546; doi:10.1371/journal.pone.0032157)
Supplement: Methods S1 — Supplementary methods. (DOCX) [file pone.0032157.s002.docx]

**Supplementary Methods**

**Immunostaining**. Confluent monolayer of HUVEC cultures was fixed with 3% PFA in PBS for 30 min, permeabilized with 0.5% Triton X-100 in PBS supplemented with 3% BSA, and stained with a rabbit anti-human VWF (Sigma) and a monoclonal mouse anti-human CD31 (Sigma) antibodies. Secondary antibodies goat Alexa 488-conjugated anti-mouse or Alexa 594-conjugated anti-rabbit were used. Nuclei were stained with DAPI. Pictures were taken using an Axiovision upright microscope and processed using Photoshop.
